# AI-based Assessment of Risk Factors for Coronary Heart Disease in Patients With Diabetes Mellitus and Construction of a Prediction Model for a Treatment Regimen

**DOI:** 10.31083/RCM36293

**Published:** 2025-06-25

**Authors:** Zhen Gao, Qiyuan Bai, Mingyu Wei, Hao Chen, Yan Yan, Jiahao Mao, Xiangzhi Kong, Yang Yu

**Affiliations:** ^1^Department of Cardiac Surgery, Beijing Anzhen Hospital, Capital Medical University, 100069 Beijing, China; ^2^Department of Cardiac Surgery, The First Clinical Medical College of Lanzhou University, 730000 Lanzhou, Gansu, China; ^3^Department of Cardiology, Peking University Third Hospital, NHC Key Laboratory of Cardiovascular Molecular Biology and Regulatory Peptides, Peking University, 100191 Beijing, China; ^4^Center for Coronary Artery Disease, Division of Cardiology, Beijing Anzhen Hospital, Capital Medical University, 100069 Beijing, China

**Keywords:** coronary heart disease, diabetes mellitus, machine learning, predictive modeling, SHapley Additive exPlanation

## Abstract

**Background:**

This study aimed to construct a prediction model for a treatment plan for patients with coronary artery disease combined with diabetes mellitus using machine learning to efficiently formulate the treatment plan for special patients and improve the prognosis of patients, provide an explanation of the model based on SHapley Additive exPlanation (SHAP), explore the related risk factors, provide a reference for the clinic, and concurrently, to lay the foundation for the establishment of a multicenter prediction model for future treatment plans.

**Methods:**

To investigate the relationship between concomitant coronary heart disease (CHD) and diabetes mellitus (DM), this study retrospectively included patients who attended the Beijing Anzhen Hospital of Capital Medical University between 2022 and 2023. The processed data were then input into five different algorithms for model construction. The performance of each model was rigorously evaluated using five specific evaluation indicators. The SHAP algorithm also provided clear explanations and visualizations of the model's predictions.

**Results:**

The optimal set of characteristics determined by the least absolute shrinkage and selection operator (LASSO) regression were 15 features of general information, laboratory test results, and echocardiographic findings. The best model identified was the eXtreme Gradient Boost (XGBoost) model. The interpretation of the model based on the SHAP algorithm suggests that the feature in the XGBoost model that has the greatest impact on the prediction of the results is the glycated hemoglobin level.

**Conclusions:**

Using machine-learning algorithms, we built a prediction model of a treatment plan for patients with concomitant DM and CHD by integrating patients' information and screened the best feature set containing 15 features, which provides help and strategies to develop the best treatment plan for patients with concomitant DM and CHD.

## 1. Introduction

In recent years, the morbidity and mortality rates of coronary heart disease (CHD) have been on the rise, with the age of onset decreasing annually [1, 2, 3, 4, 5, 6]. Meanwhile, diabetes mellitus (DM) has reached epidemic proportions worldwide, and its prevalence is also on the rise [7, 8]. CHD and DM, as two separate pathological entities, can enhance each other’s disease progression, and the mortality rate of patients suffering from both is higher than that of patients with just one [9, 10, 11, 12, 13, 14, 15, 16, 17]. Therefore, the development of treatment regimens for patients with both DM and CHD needs to take into account the common factors and influences of the two diseases [18, 19, 20, 21, 22]. At this time, treatment options for patients with both DM and CHD can be broadly divided into two categories: conservative treatment with medication after glycemic control and surgical treatment (including percutaneous coronary interventions and coronary artery bypass grafting) [23, 24]. Due to human errors and imperfections in examination and testing indicators, many patients are still unable to receive appropriate treatment plans, such that the prognosis and recovery of patients cannot be optimized [25, 26]. In recent years, machine learning has often been used to deal with this kind of data involving magnanimous samples and data mining [27, 28].

Machine learning is dedicated to the study of how computers can simulate or implement human learning behaviors to acquire new knowledge or skills and reorganize existing knowledge structures to continuously enhance their performance [29, 30, 31, 32, 33]. Machine learning can systematically process and classify much clinical data on its own and ultimately obtain information of clinical interest from the system’s output [34, 35], which can help to reveal the essential features of the disease and elucidate the potential correlation between the information of different variables [36, 37, 38, 39, 40, 41, 42, 43, 44]. In recent years, it has been shown to provide useful insights into cardiovascular diseases and has begun to have clinical applications [45, 46, 47, 48, 49, 50, 51].

In this study, we aimed to develop and validate a prediction model for treatment regimens of patients with CHD combined with DM by conducting a retrospective study using a single-center database. We used five machine-learning algorithms for model construction, from which we identified the eXtreme Gradient Boost (XGBoost) algorithm as the best algorithm and used it as a basis for mining the relevant risk factors.

## 2. Materials and Methods

### 2.1 Data Source and Study Population

The Coronary Heart Disease Database of the Anzhen Hospital of the Capital Medical University is a platform-type operation and management system for disease resource sharing customized and developed for the Coronary Heart Disease Database platform of the Anzhen Hospital on the basis of the Jiahemeikang Disease Resource Sharing Management System. In this study, we exported 3171 patients diagnosed with coronary heart disease combined with DM from the Coronary Heart Disease Database of the Beijing Anzhen Hospital of the Capital Medical University in 2022 and 2023 and retrospectively included 3153 patients with coronary heart disease combined with DM in the internal cohort after filtering out useless data that did not meet the criteria for nullclassification or with missing features greater than 30 or more items.

Inclusion criteria:

(1) Patients with a clear diagnosis of coronary artery disease (CAD) combined with DM;

(2) After rigorous history taking, important data were complete;

(3) Age ≥18 years.

Exclusion criteria:

(1) Pregnant patients;

(2) Combination of malignant tumors and long-term use of chemotherapy drugs;

(3) Combination of diseases that can significantly affect routine blood and biochemical indices.

### 2.2 Data Collection and Preprocessing

Restricted pre-processing of the collected data was performed by coding categorical variables such as heart failure, atrial fibrillation, or cardiogenic shock using 0 and 1, representing that the sample in which 0 did not have this characteristic, and 1 did have this characteristic. In addition, factorization was performed. All data were divided into positive (Group P) and negative (Group N) groups based on the presence or absence of the treatment (i.e., percutaneous stenting and coronary artery bypass grafting). All continuous variables comparing the clinical data of the two groups of patients were described using either $\bar{x}$ ± S (satisfying normal distribution) or M (Q1, Q3) (not satisfying normal distribution). Categorical variables (count data) were described using percentages and frequencies. Statistical analysis used the R language (R 4.3.2, R Core Team, Vienna, Austria) for subsequent predictive model construction and data visualization.

To make each feature in the results comparable, all the data are first standardized, and all the data was randomly divided in the ratio of 8:2, i.e., 80% of the data were used as the training set and 20% of the data were used as the test set. The screening of features, model construction, and parameter tuning were all done in the training set, and it is guaranteed that data leakage in the test set. Based on the training set data, dummy variables (DVs) were introduced for variables that did not need to be classified and then regressed by the least absolute shrinkage and selection operator (LASSO). To optimize the regularization strength of the LASSO regression model, a grid search was carried out to determine the optimal alpha value.

For missing data, features with greater than 15% missing data were deleted, and features with no more than 15% missing data were inputed into the random forest algorithm. The random forest imputation process consists of the following steps: First, for each feature with a missing value, a random forest regression model was constructed using the other features as inputs [52]. Second, simple statistics (e.g., mean or median) were used to estimate the missing values. The model is then trained in the complete case (samples without missing values) and used to predict missing values, replacing the initial imputations. This process is repeated until the model converges or a predetermined number of iterations is reached to ensure stable input results [53]. If the data are unbalanced samples, the Synthetic Minority Over-Sampling Technique (SMOTE) algorithm is introduced to eliminate the effect of imbalance before splitting the data, where SMOTE generates new synthetic samples in the vicinity of the minority class instances with the aim of enhancing their representativeness [54].

### 2.3 Model Construction

Five different machine learning algorithms, namely Random Forest (RF), Logistic Regression (LR), XGBoost, Support Vector Machine (SVM), and K-nearest neighbor (KNN), were used in this experiment.

#### 2.3.1 RF

Random forest is an integrated machine learning algorithm that improves the accuracy and robustness of a model by combining the predictions of multiple decision trees. For the classification task, the prediction result of random forest is usually obtained through the majority voting mechanism, that is, each tree gives a prediction result, and the majority category is finally selected as the prediction result of random forest. This process can be expressed as: for the input feature vector x, each tree $t_{i}$ in the random forest gives a prediction result $y_{i}$ (for classification tasks, $y_{i}$ is a category label). The final prediction class y* is the class that maximizes the following expression:

$$y*=\arg\max_{y}\sum_{i=1}^{k}I\left(y_{i}=y\right)$$

Where, I⁢()is the indicator function, which takes the value 1 when the parenthesis condition is true, otherwise it is 0; k is the number of trees in the random forest; y is the possible category label.

#### 2.3.2 LR

LR belongs to probabilistic nonlinear regression, which is mainly used to study the relationship between the outcome index of binary classification (dependent variable) and some influencing factors (independent variable) (can be extended to multiple categories). It is commonly used in epidemiology to analyze quantitative relationships between diseases and associated risk factors. The LR model can be expressed as:

$$P=\frac{1}{1+\exp\left[-\left(\beta_{0}+\beta_{1}\,X_{1}+\beta_{2}\,X_{2}+\cdots +\beta_{m}\,X_{m}\right)\right]}$$

In the formula, *P* is the probability when a positive result occurs, $\beta_{0}$ is the constant term, $\beta_{1}$, $\beta_{2}$…, $\beta_{m}$ is the independent variable regression coefficient of $X_{1}$, $X_{2}$, …, $X_{m}$. Logarithmic conversion of the formula can be expressed in linear form:

$$\mathrm{logit}P=\ln\frac{P}{1-P}=\beta_{0}+\beta_{1}\,X_{1}+\beta_{2}\,X_{2}+\cdots +\beta_{m}\,X_{m}$$

The l⁢o⁢g⁢i⁢t⁢Pfor positive results with negative results occur when probability of the natural logarithm and the value ofl⁢o⁢g⁢i⁢t⁢P ranges have no numerical bounds.

#### 2.3.3 XGBoost

XGBoost is an ensemble learning algorithm based on gradient-raising decision trees that optimizes the loss function by adding prediction trees, each attempting to correct the error of the previous tree‌. The core idea of XGBoost is to combine multiple weak classifiers (decision trees) into one strong classifier. Its mathematical formula mainly involves the definition and optimization of the objective function. The objective function of XGBoost can be expressed as:

$$\mathrm{obj}\left(\theta \right)=\sum_{i=1}n\,L\,\left(y_{i},\hat{y}\right)+\sum_{k=1}K\,\Omega \,\left(f_{k}\right)$$

*L ($y_{i}$, $\hat{y}$)* represents a loss function that measures the difference between the model’s predicted value $\hat{y}$ and the actual value $y_{i}$. $\Omega \,\left(f_{k}\right)$ represents the complexity of the k-th tree and is used to control the complexity of the model to prevent overfitting. θ represents the parameters of the model. XGBoost performs a second-order expansion of the loss function using Taylor’s formula to better approximate and optimize the loss function. By adding prediction trees, XGBoost gradually reduces residuals and improves the predictive performance of the model. Because of its efficiency, flexibility and powerful performance, XGBoost has been widely used in a variety of machine learning tasks such as classification, regression, and sequencing.

#### 2.3.4 SVM

SVM is a two-class classification model, its basic model is defined as the linear classifier with the largest interval on the feature space, its learning strategy is to maximize the interval, and finally can be transformed into a convex quadratic programming problem. The goal of SVM is to find a hyperplane:

$$w^{T}\,x+b=0$$

w is the weight vector and b is the bias term. The sample points of different classes are separated, and the distance from the nearest point (i.e., support vector) to the line is maximized as far as possible. This distance is called the margin, and the SVM attempts to maximize this gap.

#### 2.3.5 KNN

KNN algorithm is a simple and intuitive classification and regression method, namely the K nearest neighbor algorithm. The core idea is that a sample belongs to a class if most of the K nearest neighbors of the sample in the feature space belong to that class. The general flow of the KNN algorithm is as follows:

First determine the size of the K, that is, how many neighbors to choose to participate in the decision. The choice of K value has great influence on the performance of the algorithm. Then calculate the distance between the test object and all objects in the training set: Euclidean distance is generally adopted, and the formula is:

$$d\,\left(x,y\right)=\sqrt{\sum_{i=1}^{n}{\left(x_{i}-y_{i}\right)}^{2}}$$

Where *x* and *y* are two points in n-dimensional space, and $x_{i}$ and $y_{i}$ are their coordinates on the i-th dimension, respectively. According to the calculated distance, the K training samples closest to the test sample are found, which are the K nearest neighbors of the test sample. Then take a vote or weighted average and finally use the prediction category or predicted value as the output of the algorithm.

First, it is necessary to sample N times from the original data set (Bootstrap sampling method) to form a training set with the same size N (the comparison with the original data set is not completely consistent). If each sample in the data set has T attributes, t (t ≤ T) attributes will be randomly selected when the RF internal decision tree is split, and then the split attributes of the node will be selected according to some strategy, and finally a decision tree will be grown on the training set with size N. This is repeated m times, and a random forest of m decision trees is trained.

After completing the model parameter tuning, the predictive ability of each model was verified using a test machine, and the receiver operating characteristic curve (ROC) of each model was plotted, and precision, accuracy, and recall were selected, and the F1 score and area under curve (AUC) were selected as the evaluation indexes of model effectiveness. The model with the largest AUC was selected as the best model, and the Hosmer-Lemeshow (HL) was further performed to assess the degree of correspondence between the predicted probabilities and observations using the Hosmer-Lemeshow goodness-of-fit test [55]. *p*-values less than 0.05 indicate that there may be a model fitting problem, such as overfitting or underfitting [56]. We used the SHapley Additive exPlanation (SHAP) algorithm to explain the prediction model, which provides a globally consistent explanation of the model from the theory of game theory, can explain each feature output of the machine learning model at the group level as well as at the individual level, and visualize the output results to study the relative importance of each feature, in which the SHAP value of the edible oil bar graphs and scatter plots composed of summary graphs in a graphical representation, are used to illustrate the importance of individual features and their overall impact on model predictions [57].

### 2.4 Model Evaluation

#### 2.4.1 Accuracy

Accuracy is a measure of the percentage of all predicted samples that the model correctly predicts. In this study, accuracy provides an intuitive evaluation criterion to help us understand the predictive power of the model as a whole. By evaluating the accuracy, it is possible to determine whether the model’s performance on the test data meets expectations, thus providing a basis for further optimization and the calculation formula is:

$$\text{ Accuracy }=\frac{T\,P+T\,N}{T\,P+T\,N+F\,P+F\,N}$$

TP (True Positive) is the number of samples correctly predicted as a positive class, TN (True Negative) is the number of samples correctly predicted as a negative class, FP (False Positive) is the number of samples incorrectly predicted as a positive class, FN (False Negative) is the number of samples incorrectly predicted as a negative class.

#### 2.4.2 Precision

The accuracy rate measures the proportion of all samples predicted to be positive that are actually positive. In this study, the accuracy rate reflects the accuracy of the model when predicting positive classes, such as patients with cardiovascular disease. The higher accuracy indicates that the model can identify the real positive samples well, which is of great significance for avoiding false positive prediction and reducing misdiagnosis, and the formula is:

$$\text{ Precision }=\frac{T\,P}{T\,P+F\,P}$$

Where TP is the number of samples correctly predicted as positive, and FP is the number of samples incorrectly predicted as positive.

#### 2.4.3 Recall

The recall represents the percentage of all samples that are actually positive that are correctly predicted to be positive. Recall rates in this study were used to assess the model’s ability to identify positive samples, especially in high-risk patients. The higher recall rate means that the model can capture more actual positive samples, which is crucial for early detection of diseases and reducing missed diagnoses and is calculated as:

$$\text{ Recall }=\frac{T\,P}{T\,P+F\,N}$$

Where TP is the number of samples correctly predicted as a positive class, FN is the number of samples incorrectly predicted as a negative class.

#### 2.4.4 F1 Score

The F1 score is the harmonic average of the accuracy rate and the recall rate, which takes into account the accuracy and completeness of the model in the positive prediction. In this study, F1 scores provide a way to balance accuracy and recall, especially when dealing with data imbalances. With F1 scores, we are able to evaluate the classification performance of the model more comprehensively and its calculation formula is:

$$\text{ F1 Score }=\frac{2\times T\,P}{2\times T\,P+F\,P+F\,N}$$

#### 2.4.5 AUC

The AUC value is calculated by plotting the area under the ROC curve, and the calculation formula is usually obtained by numerical integration. AUC is an important index to evaluate the performance of binary classification models, which measures the ability of models to distinguish between positive and negative classes. In this study, the AUC values reflect the comprehensive performance of the model under different thresholds. A higher value of AUC means that the model can distinguish positive and negative samples more effectively, and has a strong classification ability. Especially in the case of unbalanced categories, AUC is a very useful performance evaluation standard.

#### 2.4.6 Matthews correlation coefficient (MCC)

MCC is a comprehensive index considering all classification results, which can fully reflect the classification performance of the model. In this study, MCC is used to evaluate the performance of the model in the face of unbalanced data. The closer the value of MCC is to 1, the better the prediction results of the model are. Especially when dealing with small samples of positive or negative classes, MCC provides a more stable performance evaluation and its calculation formula is:

$$\mathrm{MCC}=\frac{T\,P\times T\,N-F\,P\times F\,N}{\sqrt{\left(T\,P+F\,P\right)\,\left(T\,P+F\,N\right)\,\left(T\,N+F\,P\right)\,\left(T\,N+F\,N\right)}}$$

#### 2.4.7 Hosmer-Lemeshow Test Statistic

The Hosmer-Lemeshaw test is used to evaluate the goodness of fit of a model, and it tests the agreement between the predicted values of the model and the actual observed values. In this study, the test is used to determine whether the model can accurately fit the data and whether there are systematic errors. Through this test, we can confirm the model’s consistency across different data sets, thereby enhancing its reliability for clinical application and the statistics of the Hosmer-Lemeshaw test are usually calculated by the following formula:

$$\hat{C}=\sum_{k=1}^{G}\frac{{\left(O_{1\,k}-E_{1\,k}\right)}^{2}}{N_{k}\times {\bar{\pi }}_{k}\times \left(1-{\bar{\pi }}_{k}\right)}$$

Here, G represents the number of groups (usually 10 groups), $O_{1\,k}$is the actual number of events observed in group kk (i.e., the number of samples with the dependent variable taking the value of 1), $E_{1\,k}$ is the number of events predicted by the model in group kk (that is, the sum of the predicted probabilities of all samples in this group), $N_{k}$is the total sample size of Group k, ${\bar{\pi }}_{k}$is the average of the predicted probabilities of the k group.

#### 2.4.8 Hosmer-Lemeshow Test *p*-value

The *p*-value is calculated from the statistics of the Hosmer-Lemeshaw test, which is usually tested based on the Chi-square distribution. The *p*-value of Hosmer-Lemeshaw test is used to evaluate the fitting effect of the model, and a higher *p*-value indicates a better match between the model and the actual data. In this study, the *p*-value helped us judge the applicability of the model to clinical data, ensuring that its prediction results have a high degree of confidence in practical applications.

#### 2.4.9 Confusion Matrix

The confusion matrix intuitively reveals the classification results of the model by showing the comparison between the actual categories and the predicted categories of the model. In this study, the confusion matrix helps us to understand the predictive performance of the model for various samples, especially whether it can correctly identify positive and negative classes. With this tool, we are able to evaluate the specific performance of the model in each category and provide specific directions for subsequent improvement. The confusion matrix usually looks like this:

$$\left[\begin{array}{cc} T\,N & F\,P\\ F\,N & T\,P \end{array}\right]$$

Where TP, TN, FP and FN represent true example, true counter example, false positive and false counter example respectively.

#### 2.4.10 Calibration Curve

The calibration curve evaluates the calibrability of the model by calculating the difference between the actual incidence and the predicted probability for each predicted probability interval. Specific formulas usually involve calculating by ratios or differences. The calibration curve shows the agreement between the probability predicted by the model and the actual results. In this study, the accuracy of the model’s prediction probabilities was evaluated by calibration curves to ensure that the model was not only able to classify, but also to provide reliable probability predictions. The good calibration curve shows that the probabilistic prediction of the model is consistent with the actual incidence rate, which enhances its operability and reliability in the actual clinical environment.

### 2.5 Research Quality Control

Strict quality control is used in order to ensure the reliability and accuracy of the study: (1) the collection process of the samples is carried out by two investigators in strict accordance with the development of the inclusion and exclusion criteria, and controversial cases are discussed and resolved with the intervention of a third person; (2) the sample data collection is completed for comparison, to ensure that there are no data extraction mismatches and omissions; (3) the inclusion of the data is carried out once again before inputing the data, sample set features containing missing values are deleted; (4) data are processed using the R language, and after the code is written, the code is repeated several times to check the code to ensure the accuracy of the results.

### 2.6 Literature Search Strategy

To ensure the comprehensiveness and rigor of the literature, we adopted a multi-dimensional literature search strategy in this study, which includes the following aspects: First, we searched for literature related to the risk factors and treatment strategies specifically for diabetic and CAD populations. The focus was on the complications of cardiovascular diseases in diabetic patients, risk assessments for CAD, the impact of diabetes on cardiovascular health, the effectiveness of drug treatments, and the role of lifestyle interventions (such as diet and exercise) in the prevention and treatment of CAD. These sources provided theoretical support for feature selection and helped identify key risk factors related to both diabetes and CAD.

Next, we searched for commonly used machine learning models in clinical prediction, particularly those applied in cardiovascular disease prediction. The search included traditional machine learning algorithms, such as logistic regression, SVM, and random forests, which are widely used in clinical data prediction modeling. Additionally, we explored the application of deep learning algorithms, such as neural networks, convolutional neural networks (CNN), and Transformer architectures, and assessed their applicability, advantages, and limitations in cardiovascular disease prediction. These sources provided valuable theoretical and practical guidance for model selection and optimization.

We then conducted further searches on the risk factors and treatment strategies for diabetes patients with CAD. Specifically, we focused on the unique characteristics of diabetic patients with CAD, such as the impact of diabetes on vascular health, the comorbid mechanisms between diabetes and CAD, and the effectiveness of combined treatments for diabetes and cardiovascular diseases. These studies contributed to a deeper understanding of the risks and treatment strategies specific to diabetic patients with CAD.

## 3. Results

### 3.1 Population Characteristics Overview

In this study, we retrospectively included patients with concomitant DM and CHD who attended the Beijing Anzhen Hospital of the Capital Medical University from 2023 to 2024. We included 3153 patients after strictly screening potential participants per the nadir criteria. Among them, 2056 patients received treatment (percutaneous coronary stenting or coronary artery bypass grafting), and 1097 patients received conservative treatment.

**Fig. 1.**
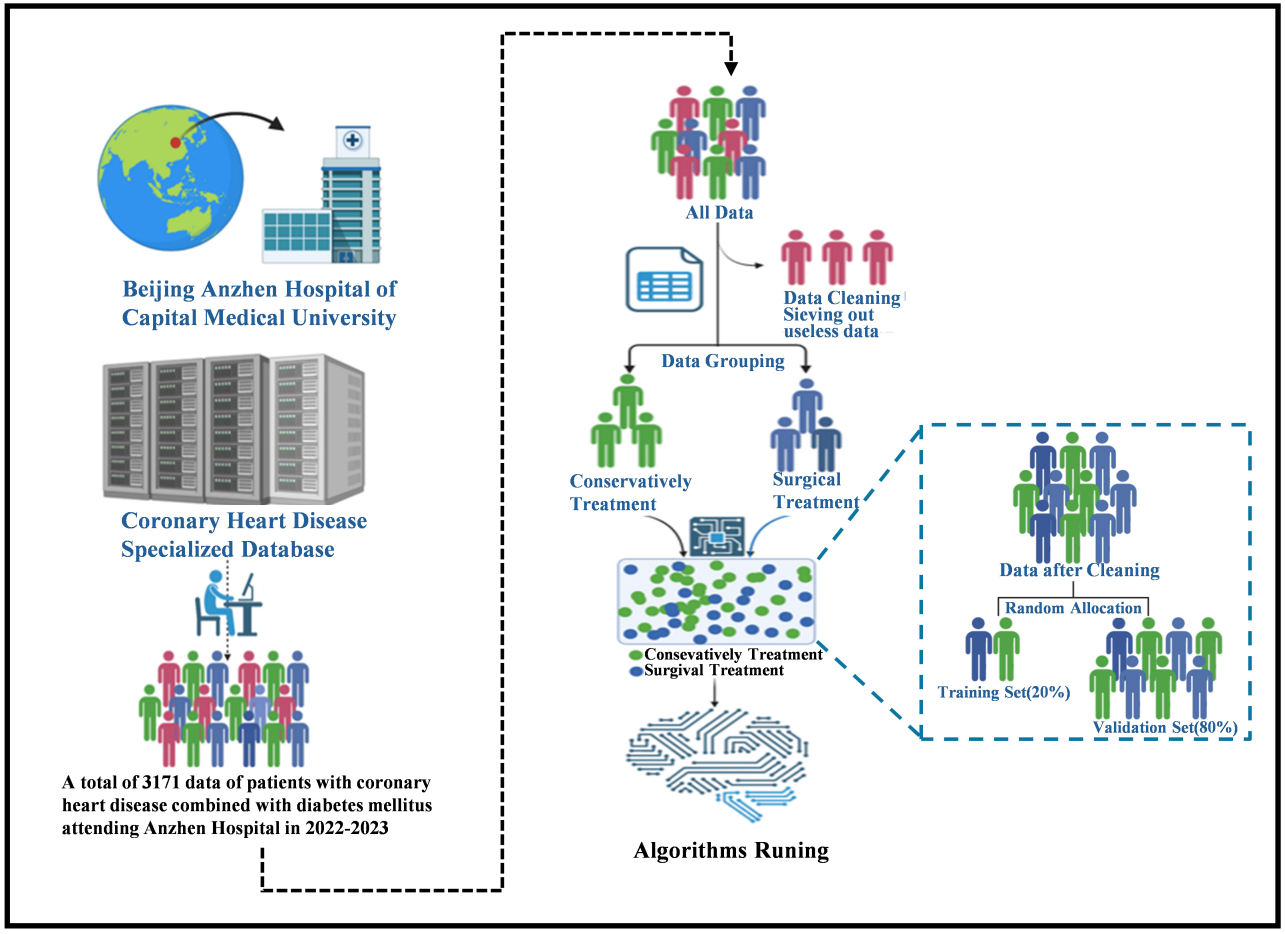
**Flowchart of AI framework establishment**. The population with coronary heart disease combined with diabetes mellitus, sourced from the Coronary Heart Disease Specialized Database of the Beijing Anzhen Hospital affiliated with the Capital Medical University between 2022 and 2023, was retrospectively included. A total of 3171 cases were exported. After cleaning, which involved removing cases that did not meet the inclusion criteria and those with more than 30 missing features, 3153 cases remained. These cases were then categorized based on whether the patients had undergone treatment (percutaneous interventional or coronary artery bypass grafting). AI, artificial intelligence.

After excluding entries containing missing values, feature screening was performed with the LASSO regression within the training set (the differences between the training and test sets were not statistically significant), and the optimal parameter alpha of 0.02 was obtained after validation through grid search, corresponding to the smallest error. The features with non-zero coefficients could be included at this time, and the final set of the best features was obtained, which included: sex, age, and the randomized glucose level, positivity or weak positivity of fecal occult blood, free thyroxine level, erythrocyte distribution width, coefficient of variation, glycosylated hemoglobin level, high-density lipoprotein cholesterol level, hemoglobin level, glomerular filtration rate, alanine aminotransferase titer, pulmonary artery trunk internal diameter, maximum left ventricular diastolic E-wave flow velocity, maximum aortic flow velocity, and hospitalization period, and activated partial thromboplastin time. This optimal feature set was used in the construction of the subsequent five predictive models. The flowchart is shown in Fig. 1.

The general data of treatment group (n = 2056) and conservative treatment group (n = 1097) were compared: the ages (years) were 62.59 ± 8.67 and 64.73 ± 9.28, respectively. There were 1527 (74.27%) and 803 (73.20%) males, respectively. Fecal occult blood was positive or weak positive in 258 (12.55%) cases and 166 (15.13%) cases. Heart failure occurred in 381 (18.53%) cases and 303 (27.62%) cases, respectively. Comparison of relevant data of laboratory examination results: the results of random blood glucose examination (mmol/L) were 6.49 ± 2.58 and 7.05 ± 2.84, respectively. The results of low density lipoprotein cholesterol (mmol/L) were 2.19 ± 0.85 and 2.26 ± 0.87, respectively. The relative data of echocardiography were compared: left ventricular ejection fraction (%) was 58.99 ± 8.71 and 58.22 ± 10.95, respectively. The maximum diastolic A-wave velocity (cm/s) of left ventricular were 89.95 ± 20.69 and 91.45 ± 24.73, respectively. The main internal diameters of pulmonary artery (mm) were 23.16 ± 2.54 and 23.6 ± 3.1, respectively. The maximum velocity of pulmonary artery (cm/s) was 90.25 ± 15.36 and 91.17 ± 18.73, respectively. The maximum E-wave velocity (cm/s) in left ventricular diastolic period were 72.21 ± 24.92 and 80.79 ± 35.42, respectively. Comparisons of general data between the positive group (n = 2056) and the negative group (n = 1097) are presented in Table 1.

**Table 1.** **Comparison of baseline characteristics between the two data sets**.

		Negative group	Positive group	Standardize difference	*p*-value
Sample size		1097	2056		
Age		64.73 ± 9.28	62.59 ± 8.67	0.24 (0.16, 0.31)	<0.001*
BMI		25.40 ± 3.30	25.71 ± 3.13	0.10 (0.02, 0.17)	0.007*
Random blood glucose test results		7.05 ± 2.84	6.49 ± 2.58	0.21 (0.13, 0.28)	<0.001*
Neutrophil count findings		4.25 ± 1.56	4.28 ± 1.53	0.02 (–0.06, 0.09)	0.368*
Total thyroid hormone results		123.29 ± 21.50	122.32 ± 22.04	0.04 (–0.03, 0.12)	0.162*
Free thyroxine (FT4) test results		11.76 ± 1.95	11.45 ± 2.00	0.16 (0.09, 0.23)	<0.001*
Fasting blood glucose test results		7.05 ± 2.84	6.49 ± 2.58	0.21 (0.13, 0.28)	<0.001*
Blood creatinine test results		90.70 ± 59.97	80.60 ± 40.24	0.20 (0.12, 0.27)	<0.001*
Lactic acid test value		1.73 ± 0.52	1.72 ± 0.62	0.02 (–0.05, 0.10)	0.006*
Activated partial thromboplastin time test result		31.75 ± 4.18	31.59 ± 4.69	0.04 (–0.04, 0.11)	0.369*
Results of coefficient of variation of erythrocyte distribution width		13.23 ± 1.05	13.02 ± 0.90	0.21 (0.13, 0.28)	<0.001*
Glycated hemoglobin test results		7.19 ± 1.01	7.03 ± 0.88	0.17 (0.09, 0.24)	<0.001*
Platelet count findings		214.39 ± 65.36	217.70 ± 56.00	0.05 (–0.02, 0.13)	0.009*
HDL cholesterol test results		1.04 ± 0.27	1.00 ± 0.24	0.16 (0.08, 0.23)	<0.001*
White blood cell count results		6.58 ± 1.82	6.63 ± 1.74	0.03 (–0.04, 0.10)	0.187*
Hemoglobin test results		136.10 ± 17.94	139.04 ± 15.39	0.18 (0.10, 0.25)	<0.001*
Triglyceride test results		1.62 ± 0.96	1.64 ± 0.98	0.02 (–0.05, 0.10)	0.120*
High Sensitivity Troponin I (HS-TnI) test results		105.75 ± 669.86	140.01 ± 998.08	0.04 (–0.03, 0.11)	<0.001*
Creatine kinase isoenzyme test results		2.07 ± 2.51	2.10 ± 3.11	0.01 (–0.06, 0.08)	0.092*
PT-International Normalized Ratio (INR) test results		1.02 ± 0.19	1.00 ± 0.16	0.11 (0.04, 0.19)	0.753*
Monocyte count findings		0.42 ± 0.14	0.42 ± 0.12	0.02 (–0.06, 0.09)	0.698*
Total cholesterol results		4.06 ± 1.08	3.96 ± 1.05	0.09 (0.02, 0.16)	0.032*
Mean platelet volume findings		9.98 ± 1.04	9.97 ± 0.97	0.01 (–0.06, 0.08)	0.695*
Homocysteine test results		16.80 ± 8.68	16.56 ± 8.34	0.03 (–0.05, 0.10)	0.302*
Apolipoprotein A test results		58.77 ± 56.87	60.85 ± 58.37	0.04 (–0.04, 0.11)	0.305*
Ultrasensitive C-reactive protein test results		3.35 ± 4.70	2.96 ± 4.28	0.09 (0.01, 0.16)	0.060*
Glomerular filtration rate		80.54 ± 21.98	86.26 ± 16.64	0.29 (0.22, 0.37)	<0.001*
Thyroid stimulating hormone test results		2.72 ± 5.61	2.97 ± 5.85	0.04 (–0.03, 0.12)	0.898*
Thyroid function TT3 test		1.41 ± 0.28	1.44 ± 0.28	0.09 (0.02, 0.16)	0.040*
Prothrombin time test result		11.52 ± 2.21	11.29 ± 1.82	0.11 (0.04, 0.18)	0.507*
Erythrocyte hematocrit test results		39.73 ± 4.94	40.47 ± 4.23	0.16 (0.09, 0.23)	0.002*
Macroplatelet ratio findings		25.79 ± 7.16	25.69 ± 6.78	0.01 (–0.06, 0.09)	0.831*
Free fatty acids (FFA) test results		0.53 ± 0.23	0.52 ± 0.25	0.03 (–0.04, 0.10)	0.103*
Free Triiodothyronine (FT3) test results		4.61 ± 0.58	4.67 ± 0.65	0.09 (0.02, 0.17)	0.148*
Aspartate aminotransferase assay value		23.40 ± 84.22	21.41 ± 16.18	0.03 (–0.04, 0.11)	<0.001*
Platelet distribution width test results		16.12 ± 0.33	16.11 ± 0.31	0.02 (–0.05, 0.10)	0.400*
D-dimer test results		308.83 ± 1582.41	184.85 ± 401.38	0.11 (0.03, 0.18)	<0.001*
Lymphocyte count test results		1.72 ± 0.60	1.75 ± 0.60	0.05 (–0.02, 0.12)	0.050*
Alanine aminotransferase assay value		22.09 ± 41.62	26.12 ± 25.03	0.12 (0.04, 0.19)	<0.001*
LDL cholesterol test results		2.26 ± 0.87	2.19 ± 0.85	0.08 (0.00, 0.15)	0.070*
Left ventricular ejection fraction		58.22 ± 10.95	58.99 ± 8.71	0.08 (0.00, 0.15)	0.153*
Interventricular septal thickness (IVS)		10.50 ± 2.07	10.42 ± 1.82	0.04 (–0.03, 0.11)	0.549*
Maximum left ventricular diastolic A-wave flow velocity		91.45 ± 24.73	89.95 ± 20.69	0.07 (–0.01, 0.14)	0.273*
Left ventricular end-diastolic internal diameter		49.20 ± 7.16	48.45 ± 5.93	0.11 (0.04, 0.19)	0.097*
Internal diameter of pulmonary artery trunk		23.60 ± 3.10	23.16 ± 2.54	0.16 (0.08, 0.23)	0.002*
Aortic sinus internal diameter		34.14 ± 4.33	33.83 ± 3.81	0.08 (0.00, 0.15)	0.283*
Posterior left ventricular wall thickness		9.32 ± 1.88	9.22 ± 1.46	0.06 (–0.01, 0.13)	0.375*
Left ventricular shortening fraction findings		31.60 ± 6.09	31.84 ± 5.18	0.04 (–0.03, 0.12)	0.474*
Maximum pulmonary artery flow velocity		91.17 ± 18.73	90.25 ± 15.36	0.05 (–0.02, 0.13)	0.871*
LV diastolic E-wave flow velocity max		80.79 ± 35.42	72.21 ± 24.92	0.28 (0.21, 0.35)	<0.001*
Left ventricular end-systolic internal diameter		33.36 ± 8.00	32.36 ± 6.15	0.14 (0.07, 0.21)	0.645*
Right ventricular anteroposterior diameter		21.74 ± 3.04	21.44 ± 2.80	0.10 (0.03, 0.18)	0.017*
Right ventricular outflow tract inner diameter		27.86 ± 3.23	27.68 ± 3.12	0.06 (–0.02, 0.13)	0.175*
Amplitude of septal motion		7.18 ± 1.66	7.32 ± 1.60	0.08 (0.01, 0.16)	0.068*
Amplitude of posterior wall motion of the left ventricle		8.54 ± 2.10	8.51 ± 1.93	0.02 (–0.06, 0.09)	0.102*
Maximum aortic flow velocity		150.50 ± 78.99	137.78 ± 55.60	0.19 (0.11, 0.26)	0.009*
Ascending aortic internal diameter findings		35.61 ± 4.42	35.34 ± 3.91	0.07 (–0.01, 0.14)	0.366*
Maximum hospitalized blood creatinine		101.03 ± 79.46	96.49 ± 61.70	0.06 (–0.01, 0.14)	0.050*
Hospitalized activated partial thromboplastin time test result		33.28 ± 18.67	34.96 ± 22.21	0.08 (0.01, 0.16)	0.503*
Gender				0.02 (–0.05, 0.10)	0.514*
	Male	803 (73.20%)	1527 (74.27%)		
	Female	294 (26.80%)	529 (25.73%)		
Whether the fecal occult blood is positive or weakly positive				0.07 (0.00, 0.15)	0.043
	No	931 (84.87%)	1798 (87.45%)		
	Yes	166 (15.13%)	258 (12.55%)		
Whether heart failure has occurred				0.22 (0.14, 0.29)	<0.001
	No	794 (72.38%)	1675 (81.47%)		
	Yes	303 (27.62%)	381 (18.53%)		
Whether atrial fibrillation occurred				0.23 (0.16, 0.31)	<0.001
	No	987 (89.97%)	1972 (95.91%)		
	Yes	110 (10.03%)	84 (4.09%)		
Whether cardiogenic shock has occurred				0.14 (0.07, 0.21)	<0.001
	No	1060 (96.63%)	2030 (98.74%)		
	Yes	37 (3.37%)	26 (1.26%)		

Results in table: Continuous variables are described by Mean ± SD, and categorical variables are described by N (%). The *p*-values have been consolidated into one column, with continuous variables marked with an asterisk (*) next to the *p*-value, and categorical variables displaying the values directly. BMI, body mass index; HDL, high-density lipoprotein; PT, prothrombin time; TT3, Total Triiodothyronine3; LDL, Low-Density Lipoprotein; LV, left ventricle.

Subsequently, all included data were randomly split into training and testing sets at a ratio of 8:2. The data were preprocessed and fed into various algorithms for model construction. Upon completion of the construction, each algorithmic model was evaluated for its performance.

### 3.2 Prognostic Implication and Predictive Performance of the Five Models

**Fig. 2.**
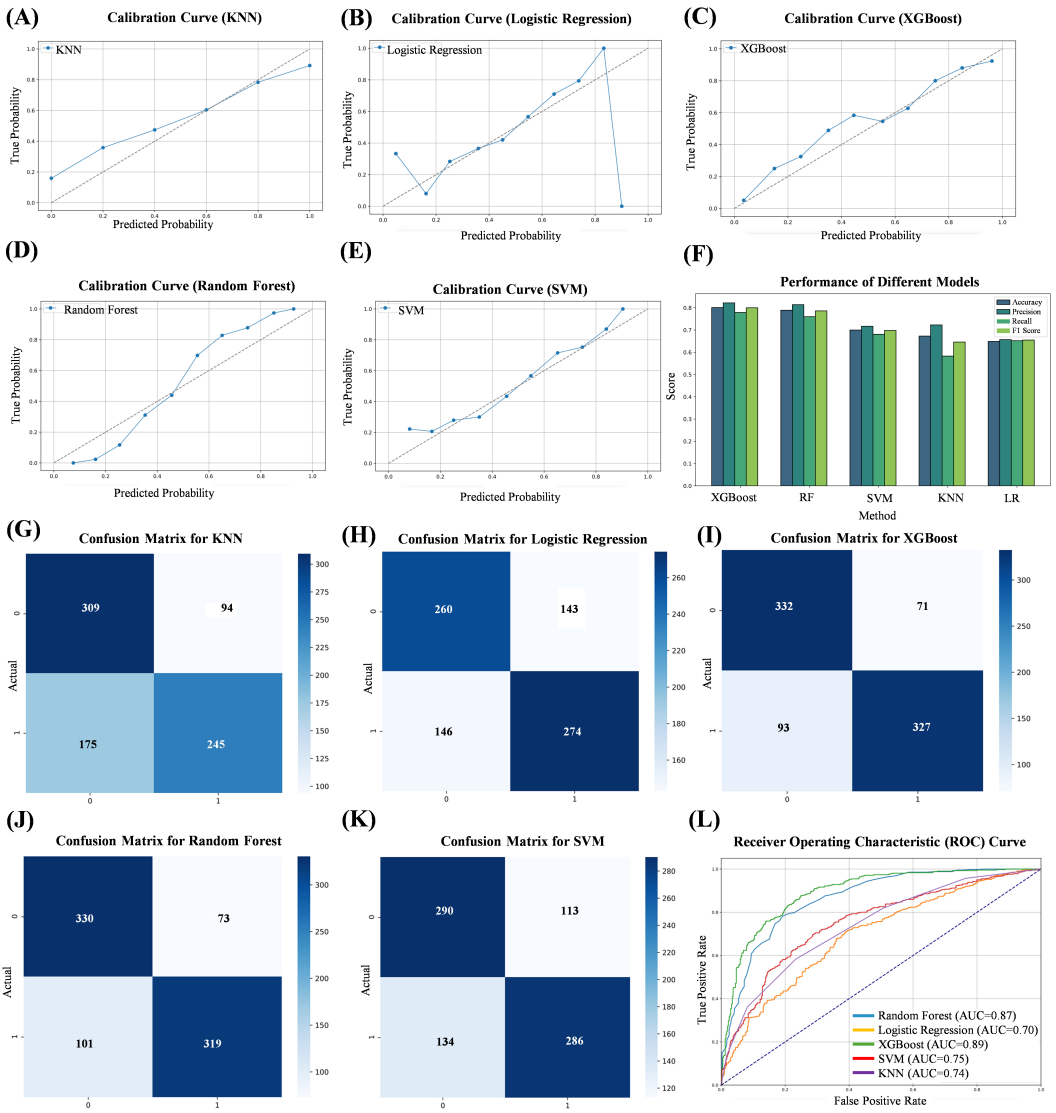
**Evaluation metrics for five machine learning algorithms**. (A) Calibration curve of the KNN. (B) Calibration curve of the LRs. (C) Calibration curve of the XGBoost. (D) Calibration curve of the RF. (E) Calibration curve of the SVM. (F) Performance of five machine learning algorithms. (G) Confusion matrix for KNN. (H) Confusion matrix for LR. (I) Confusion matrix for XGBoost. (J) Confusion matrix for RF. (K) Confusion matrix for SVM. (L) Comparison of subject work characteristics (ROC) curves for the five machine learning models. ROC, receiver operating characteristic curve.

In this study, five models were constructed based on machine learning, and the calibration curves of its five algorithms are shown in Fig. 2A–E; the ROC curves of the five algorithms are shown in Fig. 2L. Among them, the AUC of the RF model was 0.87; the AUC of the LR model was 0.70; the AUC of the XGBoost model was 0.89; the AUC of the SVM model was 0.75; and the AUC of the KNN model was 0.74. To alleviate the bias caused by the data imbalance, this study calculates additional metrics, including precision, accuracy, recall, MCC and the F1 score to comprehensively evaluate the model’s predictive performance based on the four basic metrics TP, TN, FP and FN in the model confusion matrix. Precision represents the percentage of samples that are actually positive out of all the samples predicted by the model to be positive. It measures the accuracy of the model when the prediction is a positive example, and the formula is $P\,r\,e\,c\,i\,s\,i\,o\,n=\frac{T\,P}{T\,P+F\,p}$; Accuracy represents the proportion of samples correctly predicted by the model to the total number of samples. It measures the accuracy of the overall classification of the model. The calculation formula is $A\,c\,c\,u\,r\,a\,c\,y=\frac{T\,P+T\,N}{T\,P+T\,N+F\,P+F\,N}$; Recall represents the percentage of samples that are correctly predicted to be positive by the model out of all samples that are actually positive. It measures the model’s ability to identify positive samples and is calculated as $R\,e\,c\,a\,l\,l=\frac{T\,P}{T\,P+F\,N}$; MCC is a measure of the quality of a binary classification model that takes into account all four classification outcomes (TP, TN, FP, FN) and returns a value between –1 and 1. An MCC of 1 is a perfect prediction, 0 is an average random prediction and –1 is a completely inconsistent prediction and its calculation formula is $M\,C\,C=\frac{T\,P\times T\,N-F\,P\times F\,N}{\sqrt{\left(T\,P+F\,P\right)\,\left(T\,P+F\,N\right)\,\left(T\,N+F\,P\right)\,\left(T\,N+F\,N\right)}}$; The F1 score is the harmonic average of Precision and Recall and is used to weigh between the two. It provides a single metric to evaluate the overall performance of the model and its calculation formula is $F\,1\,S\,c\,o\,r\,e=\frac{2\times T\,P}{2\times T\,P+F\,P+F\,N}$.

Based on the evaluation results of the five algorithms, it can be concluded that the XGBoost model shows better performance in all the evaluation metrics, demonstrating the best prediction. A confusion matrix (CM) is a specific table layout used in machine learning and statistics to describe the performance of supervised learning algorithms. It provides a visual representation of the comparison between the predicted and actual results of a classification model on a test dataset. The CM for the five models is shown in Fig. 2G–K. A comparison of the performances of the different models is presented in Fig. 2F and Table 2. To further assess model calibration, we used the HL test. The results, including the associated *p*-values, are shown in Table 3. RF and KNN were the poorly fitted models, whereas LR, XGBoost, and SVM were the well-fitted ones.

**Table 2.** **Performance comparison of five machine learning algorithms**.

Method	AI architecture	Data scale	Features	Accuracy	Precision	Recall	F1 score	AUC	MCC
XGBoost	Ensemble Learning (Gradient Boosting Decision Tree)	Medium	Gender, Age, Other Clinical Data	0.801	0.822	0.779	0.800	0.893	0.603
RF	Ensemble Learning (Random Forest, based on voting mechanism of multiple decision trees)	Medium	Age, BMI, Other Clinical Data	0.789	0.814	0.760	0.786	0.866	0.580
SVM	Boundary Maximization (Maximal Margin Classifier using hyperplane separation)	Medium	Gender, BMI, Other Clinical Data	0.700	0.717	0.681	0.698	0.753	0.402
KNN	Instance-based Learning (Classification based on Euclidean distance)	Medium	Gender, Other Clinical Data	0.673	0.723	0.583	0.646	0.740	0.356
LR	Linear Model (Logistic Regression based on Sigmoid function for probability prediction)	Small	Gender, Age, BMI, Other Clinical Data	0.649	0.657	0.652	0.655	0.696	0.298

AUC, area under curve; MCC, matthews correlation coefficient; XGBoost, eXtreme Gradient Boost; RF, Random Forest; SVM, Support Vector Machine; KNN, K-nearest neighbor; LR, Logistic Regression.

**Table 3.** **HL test values for five machine learning algorithms**.

	Hosmer-Lemeshow Test Statistic	Hosmer-Lemeshow Test *p*-value
Random Forest	38.739836	5.49 × 10^–⁢6^
Logistic Regression	4.048735	0.85
XGBoost	14.560336	0.068
SVM	5.828116	0.67
KNN	65.103693	4.60 × 10^–⁢11^

### 3.3 Clinical Interpretability Underlying XGBoost

**Fig. 3.**
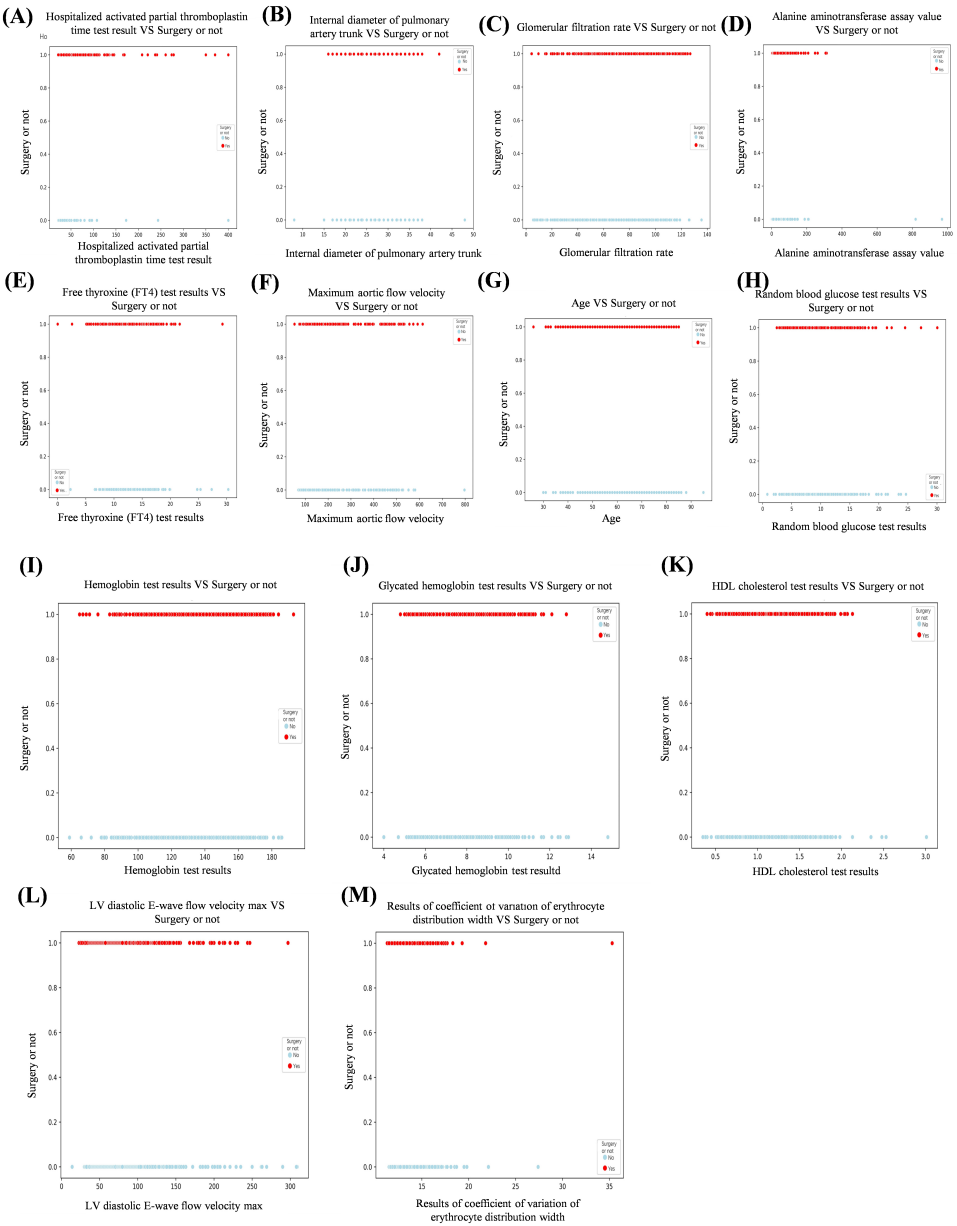
**Scatterplot of the relationship between continuous and ending variables in the features**. (A) Hospitalized activated partial thromboplastin time test results vs surgery or not. (B) Internal diameter of pulmonary artery trunk vs surgery or not. (C) Glomerular filtration rate vs surgery or not. (D) Alanine aminotransferase assay value vs surgery or not. (E) Free thyroxine (FT4) test results vs surgery or not. (F) Maximum aortic flow velocity vs surgery or not. (G) Age vs surgery or not. (H) Random blood glucose test results vs surgery or not. (I) Hemoglobin test results vs surgery or not. (J) Glycated hemoglobin test results vs surgery or not. (K) HDL cholesterol test results vs surgery or not. (L) LV diastolic E-wave flow velocity max vs surgery or not. (M) Results of coefficient of variation of erythrocyte distribution width vs surgery or not.

**Fig. 4.**
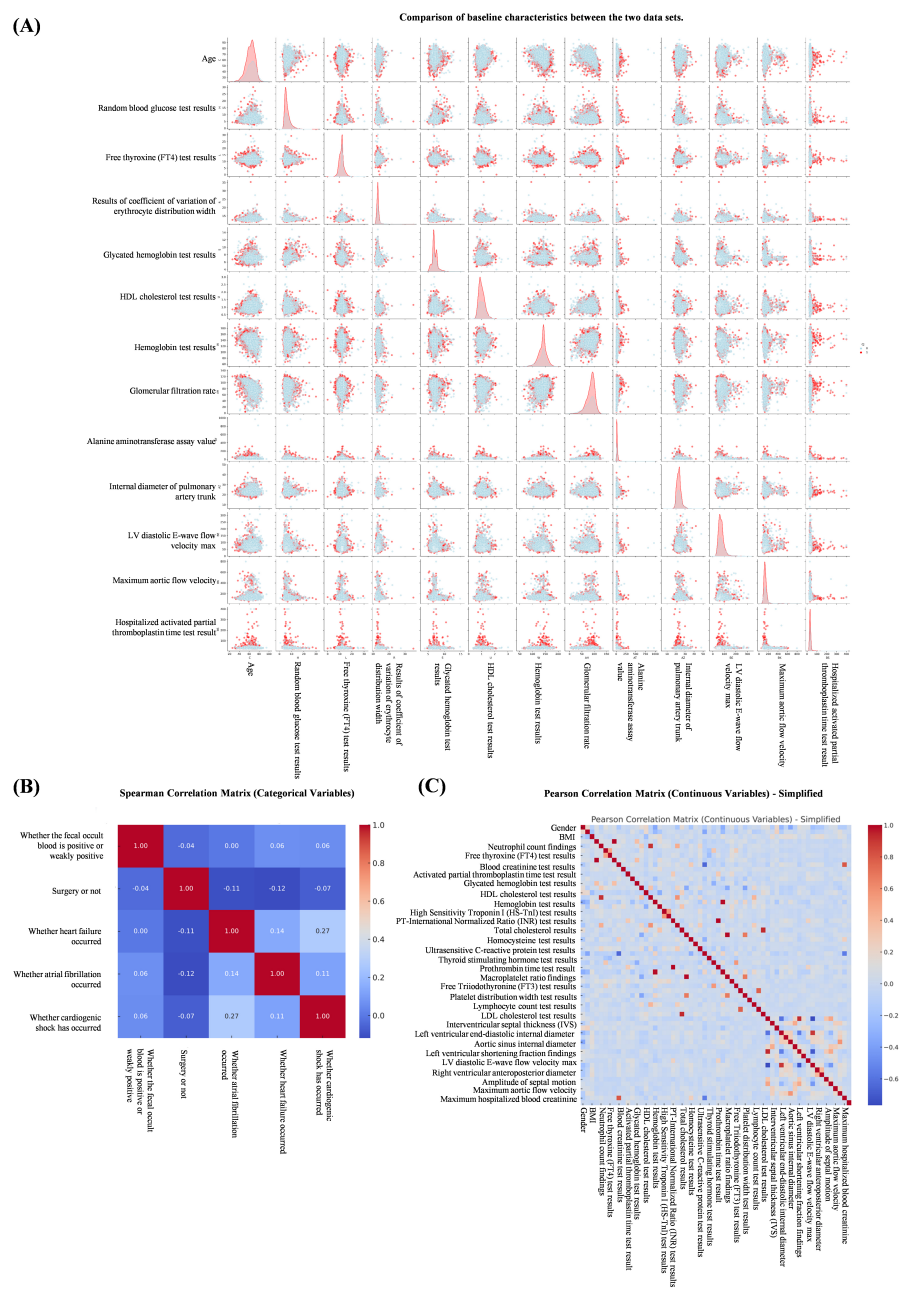
**Correlation and distribution between features**. (A) Comparison of baseline characteristics between the two data sets. (B) Spearman correlation analysis between features. (C) Pearson correlation coefficient between continuous variables in characteristics.

To visualize feature selection and treatment options and further elaborate the correlation between features, we used the XGBoost algorithm as a template to draw a scatter plot of the relationship between continuous variables and ending variables in the features (Fig. 3). At the same time, we constructed pairwise plots and Spearman correlation heatmaps using the original dataset. Pairwise plots use a color-coding system to differentiate the choice of treatment regimen, thus facilitating the observation of correlations and distributions among features (Fig. 4A). Heatmaps indicate correlations between features, with the color intensity indicating the Spearman correlation coefficient (Fig. 4B). These visualizations provide insights into the relationships between features and reveal differences in the distribution of treatment regimen choices. At the same time, we plotted the Pearson correlation coefficient, which measures the linear relationship between continuous variables in the features (Fig. 4C).

**Fig. 5.**
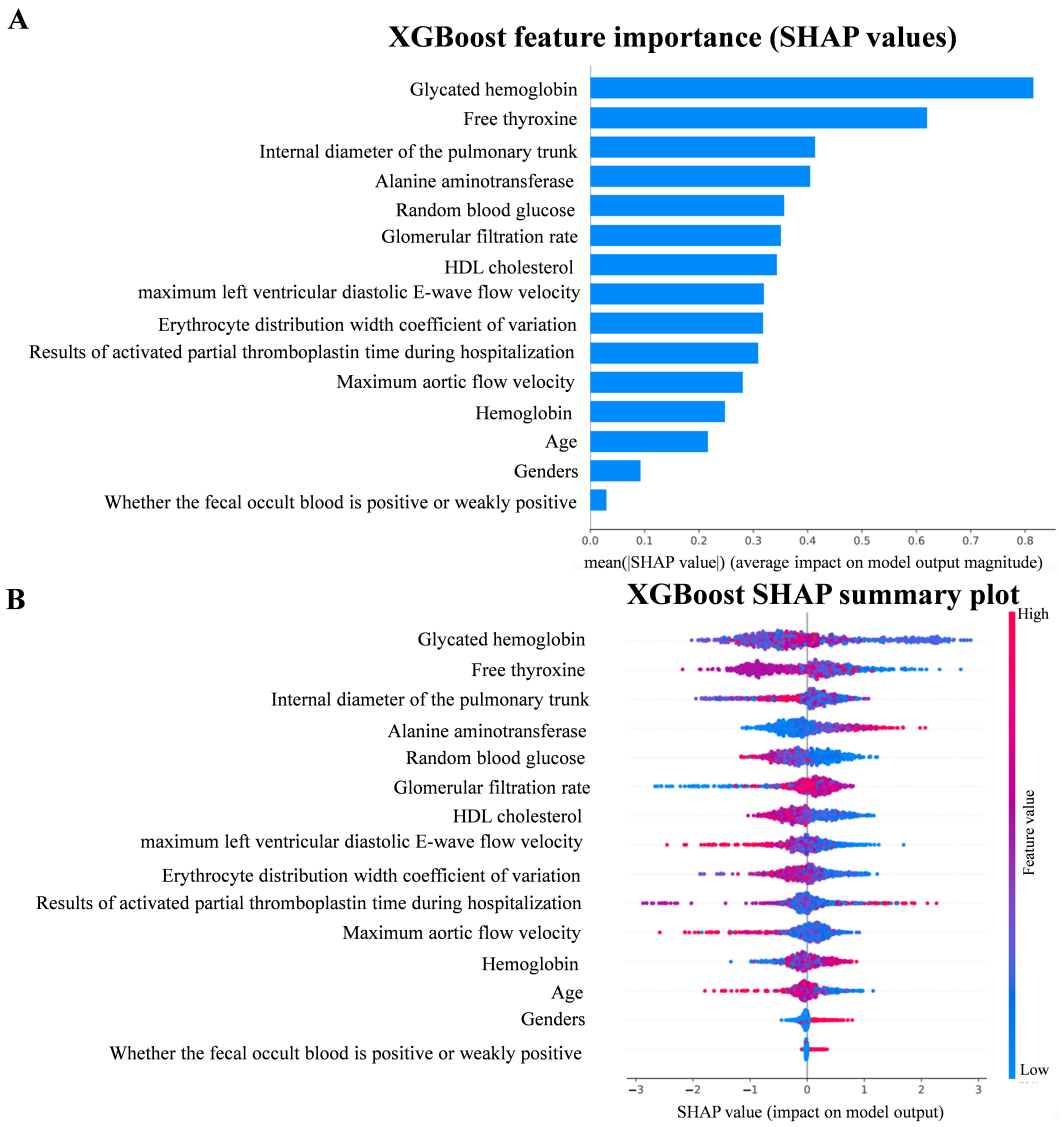
**SHAP value**. (A) Feature importance plot in XGBoost model. (B) SHAP summary plot in XGBoost model. SHAP, SHapley Additive exPlanation.

To gain a deeper understanding of the predictive power of the XGBoost model, this study used SHAP values, which elucidate the contribution of each feature to the prediction, thus enabling the identification of the key features that influence the model’s decision. To visualize the influence of the importance of each feature on the individual predictions, we used TreeExplainer from the SHAP library to calculate the SHAP values and generated SHAP summary plots and feature importance plots (Fig. 5A). From the plots, it can be seen that the feature with the greatest impact on the predictions in the XGBoost model is the glycated hemoglobin level, followed by the free thyroxine level result and the pulmonary artery trunk internal diameter. The SHAP correlation summary plots, conversely, demonstrate the impact of the XGBoost predictions on the population. The SHAP correlation summary plot shows the contribution of each feature in the XGBoost prediction model at the population level. Each point in the plot represents a sample, and the color of the point reflects the magnitude of the corresponding feature value of the sample, with red indicating that the value of the feature is relatively high and blue indicating that the value of the feature is low. Points to the right of the baseline (i.e., the dotted line) in the figure are meant to have a positive impact on the model predictions, while the opposite is true for those on the left side, with the impact increasing with the distance from the baseline (Fig. 5B).

The SHAP analysis provides a comprehensive understanding of the decision-making process of the XGBoost model and identifies relevant predictors. These findings are essential for further optimizing the model and interpreting its predictions. Meanwhile, the relevant features mined highlight their potential clinical applications, which can provide a more comprehensive assessment and a scientific basis for personalized medicine.

## 4. Discussion

In this study, we retrospectively included patients with both CHD and DM who attended the Beijing Anzhen Hospital of the Capital Medical University in 2022–2023 based on five machine-learning algorithms for prediction modeling. We found that compared with other algorithms, the XGBoost algorithm model had better predictive ability and better comprehensive performance, suggesting that machine-learning algorithms and data mining and analysis have unique advantages, which are more easily reflected in large-sample data. This algorithm is characterized by its robustness and proficiency in dealing with high-latitude features and its excellent ability to capture complex nonlinear relationships. Meanwhile, we identified 65 predictors among more than 100 features, including biological data, laboratory results, and imaging data, in which 15 features should be given sufficient attention in clinical work and provide new ideas for the treatment plan of the disease.

Age and gender became two of the 15 key factors in the predictive model for treatment options of coronary heart disease in diabetic patients, mainly due to their significant roles in physiological mechanisms, pathological changes, and treatment responses. Age influences the model through physiological changes such as increased vascular stiffness, impaired endothelial function, and exacerbated atherosclerosis with aging. These changes make elderly diabetic patients more susceptible to cardiovascular complications, requiring more cautious treatment decisions. For example, elderly patients are more likely to undergo interventional treatments (such as coronary stent implantation) rather than relying solely on medication because their blood vessels are less elastic, and the effects of medication may not be as effective as in younger patients. Additionally, elderly patients often have other chronic conditions, such as hypertension and kidney disease, which further influence treatment choices. Gender plays a crucial role due to the differences in the pathophysiology and treatment response between males and females. Men tend to exhibit more atherosclerosis and coronary artery disease at a younger age, while women experience a sharp increase in cardiovascular risk after menopause due to the loss of estrogen’s protective effects, particularly in diabetic women. Gender also affects drug metabolism and treatment adherence. Women may have different responses to certain medications (such as antihypertensive and lipid-lowering drugs) compared to men, and their treatment adherence may be lower. In summary, age and gender directly influence treatment decisions by affecting the patient’s physiological condition, disease progression, and drug response, which explains why these factors are key predictors in our machine learning model.

It is important to accurately predict the optimal treatment regimen for different individuals, as CHD and DM are two comorbid conditions with interdependent disease progressions [58, 59]. In recent years, many risk assessment and disease prognosis prediction models have been developed but not specifically for the prediction of treatment regimens in patients with both DM and CHD. A study using machine learning to predict atrial fibrillation in elderly patients with coronary heart disease and type 2 diabetes showed that the best model XGBoost had a sensitivity of 0.833, a specificity of 0.562, an accuracy of 0.587, and an AUC of 0.743, compared to existing superior models [60]. Another study of CHD diagnosis model for elderly diabetic patients based on machine learning algorithm showed that the optimal random forest model had an AUC of 0.845, an accuracy of 0.789, an accuracy of 0.778, an F1 score of 0.735, a sensitivity of 0.688, and a specificity of 0.851 [61]. Compared to these findings, our model outperforms on several key metrics: accuracy of 0.801, accuracy of 0.822, recall rate of 0.779, F1 score of 0.8, AUC of 0.893, and MCC of 0.603. These results show that our model not only makes a breakthrough in prediction accuracy, but also outperforms existing prediction models in overall performance, especially in terms of AUC and F1 scores, which further validates the effectiveness and potential clinical application value of our method. It is worth noting that in such machine-learning prediction models for predicting the risk of diabetic patients with comorbid CHD, there are the same features as in the present study, and such features show a consistent tendency and match. Sometimes, identical features have different weights in different models [62, 63, 64] suggesting that there is heterogeneity among features in disease assessment models constructed for different populations and even the same population. We also need to note that there are still barriers to the development of artificial intelligence (AI) and its integration into clinical practice [65, 66, 67], such as the need to maximize accuracy while avoiding overfitting and determine what clinical and general data should be included, taking into account convenience and the patient’s financial burden. Also, in the use of machine-learning algorithms, researchers often choose algorithms based on their own preferences and knowledge limitations, resulting in algorithms that may not be the best ones, having suboptimal prediction accuracy [68, 69]. In this regard, we utilize a standardized approach that employs retrospective studies to ensure the credibility of the basic factual results. Using multiple machine-learning models can reduce the underlying uncertainty and ultimately identify the best prediction model based on the evaluation indexes, such as the area under the AUC curve of each algorithm, accuracy, and precision, to reduce the bias of the results caused by human error.

Although this study provides valuable insights, several limitations should be acknowledged. Since this is a retrospective study, some relevant information may have been omitted during the data inclusion process. Individual cases with missing data were excluded, which resulted in a reduction of the sample size. Additionally, features with higher rates of missing values were removed, which may have led to the exclusion of potentially stronger predictive factors.

Furthermore, the model was validated solely on data from coronary heart disease patients at the Beijing Anzhen Hospital, affiliated with the Capital Medical University. It is important to note that when the model is trained on datasets with different data patterns (such as those from different hospitals, regions, or ethnic backgrounds), it may face challenges in generalizing to external populations. This can lead to incorrect predictions or an overfitting to the specificity of the training data, thereby limiting its ability to generalize to heterogeneous data not represented in the training set. To address these issues, we plan to develop related software programs or websites to support multicenter networking and improve the model’s accuracy and broad applicability.

In the future, in addition to conducting multi-center external validation, prospective validation of the model’s accuracy and universality with data from diverse and broader patient populations will be necessary. Additionally, an automatic data extraction system could be established within the database to significantly improve the efficiency of sample collection, further enhancing the robustness and scalability of the model.

In the future deployment of clinical AI models, first, the model should be integrated with existing electronic health record (EHR) systems, allowing doctors to quickly obtain real-time risk assessments and treatment recommendations based on patient data during daily care. By seamlessly connecting with clinical workflows, doctors can directly refer to the output of AI models at every stage of diagnosis and treatment, thus making more accurate decisions. Second, AI models can set thresholds and alert mechanisms to help doctors identify high-risk patients, especially in emergency or intensive care Settings, and automatically alert doctors to intervene in a timely manner. Finally, to ensure the continued effectiveness of AI models, they need to be regularly updated and optimized to respond to changing patient population characteristics and disease progression in clinical practice, ensuring the accuracy and clinical adaptability of the models. Through these measures, the proposed AI model will not only enhance personalized treatment, but also improve clinical work efficiency and ultimately optimize patient outcomes.

As the dataset continues to expand, we will further explore the application of this model in clinical settings and validate its effectiveness in real-world clinical decision-making, aiming to provide more precise and personalized treatment options for coronary heart disease patients.

## 5. Conclusion

We retrospectively included patients with concomitant DM and CHD who attended the Beijing Anzhen Hospital of the Capital Medical University in 2022–2023 based on a machine-learning algorithm and established a prediction model for the establishment of a treatment plan for these patients. We identified the XGBoost algorithm as the best one by incorporating the patients’ general information, laboratory test results, and echocardiographic findings, and screened for the optimal feature set. The optimal feature set, which contained 15 features, was selected to assist us in choosing the treatment plan. It provides help and ideas for the development of the optimal treatment plan for patients with concomitant DM and CHD.

## Data Availability

The in-house data in this study was accessed from the database of coronary heart disease within Beijing Anzhen Hospital affiliated of Capital Medical University. The in-house individual-level data is protected and cannot be shared openly due to data privacy laws, ethical restrictions and confidentiality agreements. For access to additional information required to reanalyze the data supporting the findings, please contact corresponding author (13911524101@163.com) with a detailed request and may be required to sign a data use agreement to ensure the protection of participant confidentiality.

## Abbreviations

CHD, coronary heart disease; DM, diabetes mellitus; SHAP, SHapley Additive exPlanation; DVs, dummy variables; LASSO, least absolute shrinkage and selection operator; SMOTE, Synthetic Minority Over-Sampling Technique; LR, Logistic Regression; KNN, K-nearest neighbor; RF, Random Forest; SVM, Support Vector Machine; XGBoost, eXtreme Gradient Boost; AUC, area under curve; HDL, high-density lipoprotein.
